# ‘I’m sorry to hear that’—Empathy and Empathic Dissonance: the Perspectives of PA Students

**DOI:** 10.1007/s40670-020-00979-0

**Published:** 2020-05-13

**Authors:** William F. Laughey, Megan E. L. Brown, Gabrielle M. Finn

**Affiliations:** Health Professions Education Unit, Hull York Medical School, University of York, Heslington, York, YO105DD UK

**Keywords:** Empathy, Communication, Education, Physician associate

## Abstract

**Context:**

Our understanding of clinical empathy could be enhanced through qualitative research—research currently under-represented in the field. Physician associates within the UK undergo an intensive 2-year postgraduate medical education. As a new group of health professionals, they represent a fresh pair of eyes through which to examine clinical empathy, its nature and teaching.

**Methods:**

Working with a constructivist paradigm, utilising grounded theory methodology, researchers studied 19 purposively sampled physician associate students in two UK medical schools. One-to-one semi-structured interviews were transcribed verbatim. Data were analysed using a grounded theory approach.

**Results:**

The global themes were *the pathways to empathy*, *empathy modifiers* and *empathic dissonance* a novel term to describe the discomfort students experience when pressurised into making empathic statements they don’t sincerely feel. Students preferred using non-verbal over verbal expressions of empathy. A conceptual model is proposed. The more substantial empathic pathway, affective empathy, involves input from the heart. An alternative empathy, more constrained, comes from the head: cognitive empathy was considered a solution to time pressure and emotional burden. Formal teaching establishes empathic dissonance, a problem which stems from over-reliance on the empathic statement as the means to deliver clinical empathy.

**Conclusions:**

This study furthers our understanding of the construct and teaching of empathy. It identifies empathic barriers, especially time pressure. It proposes a novel concept—*empathic dissonance—*a concept that challenges medical educationalists to reframe future empathy teaching.

## Introduction

This study explores clinical empathy from the perspective of physician assistants, or physician associates (PAs). PAs are healthcare professionals trained to the medical model. Although established for over 50 years in the USA, they are relatively new to the UK and other countries [1]. PA training programmes are an intense, 2-year journey through medical education for science graduates [2]. PA students experience a more rapid integration into clinical practice compared with their medical student counter parts.

Empathy, like other aspects of professionalism, is learned largely through the hidden curriculum [3]. In their classic paper, Hafferty and Franks indicate that medical students who are in their early years of training, and therefore most ‘lay like’, prove the most sensitive barometers of the hidden curriculum [3]. By definition, all PA students are in their first two years of training, representing a potentially insightful cohort and a fresh pair of eyes through which to view the practice and teaching of clinical empathy. Furthermore, they represent a hitherto untapped potential: there are no previous studies of empathy which focus on PA students. It is for these reasons, and underlined by the belief that the understanding of clinical empathy stands to benefit from a multidisciplinary approach, that we chose to focus on PA students for the purpose of this study.

Empathy is a pillar of patient-centred consulting [4]. When patients sense empathy from their clinician, they report greater satisfaction with the consultation; they are also more concordant with treatment and likely to enjoy better health outcomes [5, 6]. Whilst its humanist importance is not in doubt, empathy presents a series of not inconsiderable challenges to researchers and educators alike. As a complex and multi-faceted construct, it is difficult to define. Most definitions will utilise cognitive, affective or action components but there is considerable disagreement around the relative importance of these—for example, some definitions omit to mention an affective element altogether [7].

Educators face the paradox that despite efforts to teach it, there is research to suggest empathy actually declines in medical students, especially from the third year onwards [8, 9]. More recent evidence suggests this decline may be specific to some geo-sociocultural settings, being more generally observed in Western studies in the USA and UK (this study is UK based) [10]. However, the entire endeavour of measuring empathy is not without challenge or controversy. Most empathy scales rely on self-reporting, which may be unreliable, and few are validated against patient experience [11]. Qualitative studies, which could further refine our understanding of empathy, are outnumbered by quantitative papers. Pedersen’s extensive review of 209 publications [12] contained only 33 qualitative studies and, in 24 of these, empathy was peripheral to the main focus of the study. Given these limitations, researchers have called for studies to shed greater light on the nature and mechanisms of clinical empathy [12, 13], to better understand the empathic influences that students are exposed to, which this study aims to do. The research question guiding this study is as follows: *how do PA students characterise the practice and teaching of clinical empathy?* In exploring this question, our objectives are to refine our understanding of how clinical empathy is practised and taught, to propose a model to outline this and to suggest practice points for educators.

## Methods

This qualitative study is based on constructivist version of grounded theory [14]. We chose this approach as it is well-suited to the exploration and conceptualisation of complex constructs such as emapthy [14].

The researchers are two medical doctors (WL and MB) and one Professor of Medical Educational (GF). The settings are two UK medical schools, Hull York Medical School (HYMS) and Sheffield Medical School, running PA courses since 2016 and 2015 respectively. Institutional ethical approval was gained (Approval Ref: HYMS 1818). Two centres were chosen to increase the potential recruitment pool and improve the transferability of results. Inevitably, much of the existing empathy research that informs our study is based on medical students and doctors and there are likely to be significant differences between these groups and PA students, in terms of both professional identity and career trajectories. Cognisant of this, in attempting to minimise such differences, we chose two centres which educate PAs within the medical school (some UK PA courses are in universities that don’t have medical schools). Furthermore, as both courses are relatively new, being established within the last 5 years, and as PA is a relatively new profession in the UK, the PA students were subject to very few PA role models, the great majority of their tutors and supervisors were medical doctors—they were much more frequently witnessing physician rather than PA empathy. In this sense, even though we were researching PA students rather than medical students, we were none-the-less interviewing a group of students who could inform us of the influences that medical school has on empathy. Sampling was purposive, targeting PA students undertaking regular clinical placement sessions to ensure they had the opportunity to observe empathy in practice (years 1 and 2 at HYMS and year 2 in Sheffield). Recruitment was through email, posters and word of mouth.

Two researchers (WL and MB) conducted one-to-one interviews with 19 students (14 from HYMS and 5 from Sheffield) using semi-structured question stems and an iterative approach—questions evolved as data were analysed. For example, it became apparent after the first few interviews that the influence of time was seen as a key factor, and the questions were adapted to accommodate this. Question stems (Table 1) were kept as open as possible but were informed by current literature. Interviews were conducted face-to-face, or via online video or telephone depending on student preference. Interviews were audio recorded and transcribed verbatim. Focus groups were considered, but given the confidential nature of patient encounters and the potentially sensitive subject of discussing negative supervisor role models, one-to-one interviews were preferred.

**Table 1:** Question stems

Question stems	
Tell me about the empathy you see in practice…	
• Does it match your expectations?	
• Expand on thoughts, why/ why not?	
Re empathy role modelling	
• Any positive examples? Expand…	
• Any negative examples? Expand…	
Re how empathy is practiced	
• Thoughts on ways you see it done… verbal/non-verbal; affective/cognitive? Expand	
• How do you like to practice it?	
Re formal empathy teaching	
• What are your thoughts about the teaching & assessment of empathy?	
• Comment on the teaching of empathic statements	
Anything else we should discuss?	

With the intention of enhancing the analysis by ensuring multiple researchers had a close familiarity with the data, two researchers (WL and MB) independently coded all transcripts, assigning individual inductive codes to text segments on a shared file. The iterative approach to interviewing, coding and analysing allowed researchers to judge when theoretical sufficiency occurred [15], the point at which new codes were seldom required and the collected data were held sufficient to answer the study questions. In line with the iterative approach, codes were refined and sub-divided as data were simultaneously collected and analysed.

Through a series of discussions amongst all researchers, coded data were organised utilising constructivist grounded theory methodology into a three-tier structure of open codes, categories and themes [14]. Our approach was inductive but as medical educators, we were familiar with the empathy literature and literature around the hidden curriculum [3] and professional socialisation [16], concepts which informed data collection and analysis. In particular, although clinical empathy lacks any universally accepted conceptual model, there is general agreement that it comprises cognitive, affective and behavioural components [4, 7]. This was a sensitising concept for data collection and analysis, providing the broad conceptual framework for exploring empathy—it therefore informed our questions and analysis. Constructs such as cognitive and affective empathy were therefore sometimes introduced by researchers during interviews, which merits consideration when interpreting the results.

Through reflexive group discussions, all researchers considered individual preconceptions. Through conversation, the two clinicians (WL and MB) came to recognise their personal characterisations of clinical empathy did centre on emotional resonance with patients and the practice of empathy with an affective component—we recognise this will have influenced the analysis. Member checking of results with student participants was considered but rejected in line with thinking that it better aligns with positivism rather than the interpretive, constructivist approach employed in this study [17]. By contrast, triangulation with the intention of deepening the analysis was felt to be in line with the paradigm of constructivist research [17] and was facilitated through email and face to face discussions of study results with PA faculty clinical tutors at HYMS.

## Results

### Demographics

Fourteen student PAs were female and five were male. The mean age was 25, (range 22–32 years). Nine participants had previous paid healthcare experience, with the most commonly held position being that of a healthcare assistant. Fourteen participants identified as being ethnically White British, one as Black British, one as Black African and three as being from mixed ethnic backgrounds.

### Triangulation

Of the five clinical tutors approached, none expressed any disagreement with the results and two provided more in-depth verbal feedback which is tackled in the “Discussion” section of this paper.

### Overview

Initial coding generated 29 open codes which were subsequently categorised under 6 sub-themes which themselves were organised under 3 themes (Table 2).

**Table 2:** Themes, sub-themes and open codes

Theme One: Pathways to empathy	Theme Two: Empathic modifiers	Theme Three: Empathic dissonance
**Non-verbal expression conveys more than empathic statements** *Empathic statements* *Listening and questioning* *Non-verbals* *Actions & solutions*	**Empathy is proportional to time** *Busy settings* *Explanations* *Seniority & time*	***Medical school promotes forced, false empathic statements*** *Forcing empathic statements* *OSCE empathy* *Fake doesn’t convince*
**Head-heart balance** *Aspire to heart* *Witness more cognitive* *Cognitive less authentic* *Heart for serious* *Cognitive with statements* *Cognitive saves time* *Cognitive saves burden*	**Empathic alignment is aided by familiar or serious problems** *Relatable* *Serious* *Patient agendas*	**Role models** *Breaking bad news* *Negative experience leaves deeper impression*

Sub-themes are in bold and open codes in italics

The global themes were *empathy pathways*, including heart and head approaches, *empathy modifiers*, including time and relatability, and *empathic dissonance* which relates to the discomfort students feel when making statements of empathy that they themselves know to be insincere.

Results are presented under the global and organisational theme headings (Table 2):

## Global Theme: the Pathways to Empathy

### Non-verbal Expression Conveys More Than Empathic Statements

Students specified a variety of ways in which empathy could be shown to patients. Whilst they recognised that empathic statements are predominant in the teaching and practice of empathy, they themselves expressed a preference for non-verbal or problem-solving approaches. When empathic statements like ‘I am sorry to hear that’ were used, they were felt to be more effective when supported by non-verbal communication:

‘And I think maybe your tone of voice, or your non-verbal communication when you’re saying it, is also equally as important as what you’re saying.’

The act of listening was closely linked to empathy. Attentive listening to explore a patient’s problems was seen as a more effective empathic strategy than just making a quick statement and moving on:

‘Instead of saying just a statement, you could just sit, give lots of non-verbal cues and listen…’

Specific non-verbal attributes of empathy identified by students included eye contact, open body language and mirroring:

‘When a patient is really distressed, or they’re in agony… I don’t smile all the time, I try to keep my tone where the patient’s is…’

Simple actions like passing tissues, or getting down to a bed-bound patient’s height, were appreciated as empathic. More complex actions like problem-solving and finding therapeutic solutions were also seen as practical, empathic examples:

‘One of the registrars … physically came down to their level and also just spent as much time as possible going over the treatment plan…’

‘Now I can see that empathy is a lot more about thinking about what is best for someone… like choosing the best therapy for someone…’

### Head-heart balance

Although the terms cognitive and affective empathy were introduced by the researchers, students had no difficulties in agreeing that they witness both kinds. Students generally aspired to affective empathy, usually believing themselves to be a ‘heart empathy’ people. In contrast, they mostly observed cognitive empathy in practice, which felt more mechanical or robotic:

‘… probably more from the head. If it’s not from the head then there’s no empathy at all. I know it sounds awful… I think it’s either it comes from the head and it’s very mechanical or there’s no empathy at all.’

Students reported they could switch between a heart and head approach, using the head when they felt it was sufficient to act the empathic role and engaging the heart when they felt the patient had a more serious problem:

‘… so they come in with something and you’re like ‘you’re fine, I don’t think you have a problem’ but to them it is a really big problem. So you have to play the empathetic role, so I guess that’s the cognitive one there then. But when… they’ve got this really heart wrenching story you do really feel for them… then I think it becomes… from the heart.’

Although heart empathy was seen as more human and genuine, it was also recognised that it brought an emotional burden and risked the problems of compassion fatigue and a sense of bringing the patient’s problems home with you:

‘… if it all came from the heart, your empathy, then you’d be quite affected by it… I think you have to leave work at work… you can’t throw all your emotion into everything.’

Cognitive empathy was observed to be practised, not always convincingly, through the use of empathic statements:

‘…definitely the cognitive type from what I’ve seen in primary care, due to time constraints. People, as doctors, kind of just want to say, ‘I feel so sorry’ and move on to the next question.’

There was a sense in which students felt there was a level of artifice in the practice of cognitive empathy, or at least a strategy to emotionally detach from the patient. Clinicians were characterised as wearing a ‘mask’ of caring, and seen to be building walls between themselves and patients to shield them from emotional engagement:

‘I feel like as a clinician you kind of have to put on an exterior, an emotional exterior… I think that is why a lot of doctors do say the blanket statements, so it stops them from being too involved emotionally.’

‘Yes, I think a lot of doctors that I have seen giving bad news or seeing really ill patients just put up this wall… I was quite shocked by how matter-of-fact doctors were, when they were giving bad news…’

## Global Theme Two: Empathic Modifiers

### Empathy Is Proportional to Time

Time was frequently cited as a barrier to empathic practice, especially in busy setting like emergency departments, general practice surgeries and ward rounds. Breaking from ward rounds to spend time with patients signalled strong empathic intentions:

‘it was a busy ward round and the nurse… after we saw this patient who was refusing to drink his lactulose… she was like ‘you guys go, I’ll catch up with you,’ and she stayed with him for ages… put the effort into making him comfortable.’

Spending time explaining procedures, like venous cannulation, was also seen as empathic:

‘… procedures such as putting a cannula in… because it becomes such, like, a natural thing for clinicians to do they often forget to say, ‘this is what I’m doing… it might hurt a little… sorry’ kind of thing.’

Senior doctors were often seen as pressed for time and the picture emerged of juniors returning to apply empathic remediation in their wake:

‘It is probably more the higher-level people that you see lack that little bit of empathy… they’re so busy they haven’t just taken that extra two minutes to explain…. But then there was like junior doctors and they took that time to kind of go back… and show empathy.’

Students recognised that they themselves were under fewer time constraints and that the pressure of time could potentially make them less empathic in the future:

‘… how do you sort of lose that empathy or how have you lost wanting to help others? But then I haven’t got the time constraints…’

### Empathic Alignment Is Aided by Familiar or Serious Problems

Students reported it was easier to feel empathy for patients who had predicaments that they could personally relate to:

‘I think it depends if you can genuinely relate to the patient’s feelings…. If they are talking about a family member or a close friend who has passed away from cancer… I have gone through that…. it would probably be more of a heart felt thing.’

Even in more grave situations, relatability was deemed important:

‘… where someone’s had an overdose…. and maybe they’re going to pass away and I wouldn’t feel as emotional because it’s not something that I might necessarily go through.’

Students described finding it easier to feel empathy for patients facing serious diagnoses, like cancer, and conversely finding it difficult to empathise with patients who were worrying about trivial issues, like headaches. Some recognised the utility of trying to show empathy when the situation felt minor, even if it wasn’t heart-felt:

‘the patient was in tears over the cat… the professional… he was amazing because I was thinking to myself ‘I can’t really identify with her crying so much over a cat’ but he seemed to have gotten it. But when the patient walked out, you know, he laughed, so I’m like ‘well, that was false empathy’.

Unhelpful patient agendas were seen as significant barriers to empathic alignment, particularly in the arenas of drug and alcohol misuse. Students witnessed direct, harsh communication from doctors in this context, putting them in conflict with patients:

‘The doctor just simply said ‘you’re not getting any more morphine…’ and the patient was so aggressive, he started getting up trying to punch the doctor, and I was in the room, I was scared for my life.’

## Global Theme 3: Empathic Dissonance

### Medical School Promotes Forced, False Empathic Statements

Opinions around teaching centred on empathic statements. Students found these problematic, largely because they felt pushed to make these statements at times when they were not feeling for the patient, setting up a disconnect between the giving and the feeling of empathy—an empathic dissonance. This was evident both in clinical placement teaching and more formal teaching and assessment settings, especially OSCE settings:

‘I’ve been pulled up a few times by my GP because sometimes I don’t say ‘oh, I feel sorry for you’… because I feel like it’s too robotic, like the patient will think ‘you don’t really feel sorry.”

‘In one of the mock OSCEs… I got sent some negative feedback because I never showed empathy to somebody, but in that situation I wouldn’t have shown empathy to them anyway…’

OSCE empathy was singled out as being particularly likely to be lacking in true feeling, an exercise in ‘box ticking’ rather than true connection. Neither was OSCE empathy, centred almost entirely on empathic statements, felt to reflect how empathy would be demonstrated in real life:

‘Especially in the OSCE settings, I think it [the empathic statement] sounds very false because it’s something you are saying because you have to tick the box… in primary care if I see a patient myself I know I’ll be more empathetic than just saying ‘oh, I’m sorry to hear that’ … I wouldn’t just pass a comment and move on…’

Students felt forced into communicating in a less than genuine way and worried such deception would be detected by the patient:

‘… you can be speaking to someone and just be like ‘oh yeah, I’m really sorry to hear that’ but do you actually mean it? Are you sorry to hear that? And these people aren’t stupid… they can tell… if you are not being genuine, basically, it just comes across as really fake.’

### Role Models

Students reported multiple role-model empathy scenarios, especially in the arena of breaking bad news. On the one hand, positive examples included setting time aside to explain in a caring way and offering extra support from team members like specialist nurses. Positive examples were outweighed by negative ones, some doctors delivered bad news with an abruptness that seemed heartless. Interestingly, negative role model scenarios arguably left deeper educational impressions because they left students feeling degrees of psychological and emotional discomfort in terms of the gap between the empathy they expected to see and the lack of it they actually observed, such narratives were often qualified by students reflecting on deficient practice and determining not to follow in the footsteps of negative role models:

‘[the patient] was on a trolley… and he just told him…’you have lesions on your spine’, the patient had no clue what that was… there was no plan … there wasn’t much of …‘have you got anyone to speak to at the moment?’ … the guy just thought it’s because he had been carrying his bags on the wrong shoulder… I thought, ‘What’s this? Surely you could offer him a bit more empathy.’

‘I think time and time again I've seen bad, what I would say is bad communication and lack of empathy and unfortunately I think it is a problem everywhere. But I think, for me, it's sort of just driven, driven me to be the opposite. So to improve on it as it were.’

### Towards a Model of Clinical Empathy

**Fig. 1:**
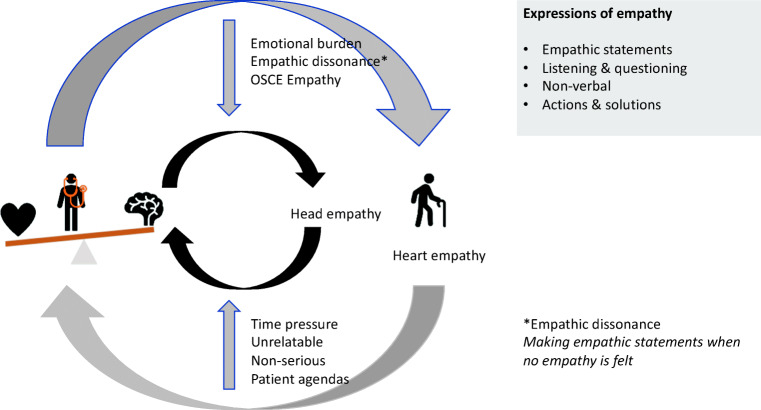
Empathy: heart and head—a conceptual model. A number of limiting factors reduce heart (affective) empathy to head (cognitive) empathy, including lack of time, difficulty relating to a patient or thinking their problem is trivial. Cognitive empathy is also seen as a solution to the emotional burden that affective empathy can bring. Cognitive empathy is associated with empathic dissonance, making empathic statements when no real empathy is felt—current teaching and assessment is fueling the problem of empathic dissonance

It was never our intention to use this study to define empathy, but given the utility for grounded theory to help in conceptualisation of complex constructs, we have used our data to propose a model which furthers our understanding of how clinical empathy is practiced and the factors which influence this (Fig. 1). The more substantial empathic pathway involves input from the heart—or at least from a balance of head and heart—an affective empathy practiced with feeling. An alternative empathy, more constrained, associated with levels of artifice, originates purely from the head and is practiced without real feeling. Affective empathy is linked with patients who have more serious problems and patients who are easy to relate to. Purely cognitive empathy is seen as a solution to the pressures of time and as a way of avoiding emotional burden. In practice, students witnessed mostly cognitive empathy. Formal teaching and assessment were seen to establish empathic dissonance, the problem of making empathic statements when no actual empathy is felt—especially true for OSCEs, a setting in which empathy was often viewed as insincere.

## Discussion

### Limitations

Before considering how these results resonate with the existing literature base, a degree of caution is required in making comparisons with medical students and physicians around whom the majority of current clinical empathy research is based: although these PA students were taught to the medical model, and tutored in medical schools and on placements shared with medical students, they are none-the-less aspiring to be a different type of professional: we believe there are more similarities than differences, but differences there undeniably are. Other factors specific to this cohort also need consideration. There is a female preponderance within the sample. Whilst this reflects the gender ratios within the PA student cohorts, it may also bias the results. There is some evidence to suggest female clinicians are more empathic than their male counterparts [18]. Although there was diversity within the study group, almost three quarters of the sample identified as White British and all three researchers are also White British: the practice of empathy has ethnic and cultural considerations [19], limiting the transferability of these results.

### Context Within the Literature and Implications

Empathy of course is not merely a preoccupation for the clinician; research around it is also rooted in social science, neuroscience, psychology and philosophy. For a construct with such broad disciplinary horizons, it would be a disservice to characterise it in terms of a simple dichotomy between head and heart—it is more nuanced than that—but definitions of clinical empathy undeniably polarise around the degree to which it is a cognitive or affective endeavour [20]. The cognitive approach is rooted in the traditional wariness of the medical profession to engage emotionally with patients, encapsulated by Fox and Lief in the philosophy of ‘*detached concern*’ [21]. Detachment is deemed necessary to avoid the clouding of logical judgment and the problem of compassion fatigue. The evidence for the latter is in actuality, conflicting: some research suggests the most empathic doctors are at the greatest risk of emotional burnout [22, 23], whilst other research indicates the most empathic clinicians are also the most resilient [24]. Proponents of the affective approach argue that empathy without feeling is no kind of empathy at all. The word itself originates from the German word ‘Ein-fühlung’, meaning ‘in feeling’. Rather than detachment, emotional resonance is advocated as the means to a sincere empathic practice [20]. Larson and Yao, conceptualising empathy as a type of ‘emotional labour’, invoke a theatrical metaphor to illustrate the difference between the two approaches, likening cognitive empathy to surface acting and affective empathy to deep acting, a method used by actors to feel emotionally as their character would, and to use the emotions to drive the performance [25].

Certainly, many of these arguments are reflected in the results of this study. PA students acknowledge the perils of burning out; reporting cognitive empathy is used as a potential solution to this. Students also see cognitive empathy as a solution to the pressures of time. There is remarkably little in the literature about empathy and time, but primary care research indicates more time leads to higher patient-perceived empathy in GP consultations [26]. It is also clear, however, that students see an affective component in empathy, the lack of which is noticed in sensitive situations like breaking bad news. As our conceptual model (Fig. 1) illustrates, students in this study see affective empathy as a more widely encompassing process, practised not just with words but with compassionate actions. By contrast, cognitive empathy has a narrower compass and is associated with levels of artifice, of ‘playing’ the empathic role, and playing it with surface rather than deep acting [25]. Students in this study aspire to affective empathy. Yet, remarkably, definitions of empathy sans feeling are commonplace in medical literature. In one review, only a quarter of studies that defined empathy included a feeling dimension and some specifically excluded it^13^. Our results challenge this, and our message to educators is to avoid teaching a purely cognitive empathy, for that is to limit the concept.

If, as reported in these results, students are witnessing mostly cognitive empathy, could this account for the finding that, in some countries at least, empathy generally declines in medical students as they progress through school, especially from the third year onwards^8**,** 10^? Are we socialising students into a kind of empathic practice that is ultimately impoverished, less natural than the one they came to medical school with? Others have suggested this may be the case [27] and the data here lends weight to this. Such adverse socialisation may not be inevitable, however. This study points to students taking positive messages from negative role-modelling—‘I don’t want that to be me’, was the reaction from some, a reaction also reported in qualitative research with medical students [28]. Educators may be able to build on this instinct, for example by asking students to reflect on how they may have done things differently when they see examples of less than adequate empathy in practice.

The empathic statement—a brief statement of empathy like ‘I am sorry to hear that’—is central to contemporary communication teaching and practice, so much so that one model of the consultation, the four habits model, recommends clinicians should strive to make an empathic statement at least once in every patient encounter [29]. However, this study suggests that there are limitations as well as merits to the empathic statement. PA students clearly see these statements as something of a paradox, serving both as a means to communicate empathy and also as a way to avoid it—a substitute for emotional engagement. Furthermore, the key reported difficulty with formal teaching and assessment is the perceived push from educators to persuade students to make empathic statements at times when no true empathy is felt, especially in OSCE assessments. PA students freely admit to practising ‘tick-box’ empathy to satisfy OSCE marking requirements.

We use the term *empathic dissonance* to capture the problem of tick-box empathy and the disconnect that occurs when students feel pressure to make statements with no true feeling behind them. The term acknowledges students’ unease around using empathic statements in this way, setting up a kind of mental conflict: feeling they should say it, but knowing they don’t mean it and worrying that patients will see through it. This echoes the mental discomfort associated with the psychological concept of cognitive dissonance [30].

If current medical training is encouraging students to fake their empathy—and these data suggest it is—then does this present a problem? There is very little research on the impact of hollow empathic statements, but qualitative research in simulated patients suggests that the insincerity in these statements is easy to detect [31]. Even if students and clinicians can learn to simulate empathy in a way that convinces patients, there remains the problem that the students and clinicians themselves know they are being less than genuine in their approach, which could inversely impact on professional satisfaction [25]. Although others have written about fake empathy [25, 27], this is the first paper to encapsulate the problem it causes to students in terms of an empathic dissonance. We see it as a symptom of over-reliance on the empathic statement at the cost of more natural empathic communication, especially non-verbal communication. Indeed, students in this study often preferred non-verbal approaches, aligning with research in psychotherapy linking empathy with effective listening [32].

If we are to address the problem of empathic dissonance, we first need to rethink how empathy is assessed in medical education. We question whether it should be assessed in OSCEs in any form, but if it is, then would it be better to reserve it for a limited number of specific stations, like breaking bad news? Students would then feel less pressure to manufacture empathic statements for every station. We also need to reframe our teaching of empathy and its bias towards the empathic statement. We don’t mean to imply that such statements are unhelpful *per se*; they can clearly be effective in conveying empathy when sincerely felt. However, they are not the beginning and the end of empathic communication, and to over-rely on them is problematic.

This data reminds us that empathy is contextual. Many students readily assume that patients with trivial presentations require little or no empathy. It’s an assumption that educators may wish to challenge. After all, one person’s mild headache is another’s feared brain tumour. Perhaps, the route to challenge is not through saying ‘show more empathy’—that is only to repeat the mistake of driving empathic dissonance—but rather to encourage what Halpern [33] terms compassionate curiosity, encouraging students to holistically explore the human perspective, including ideas, concerns and expectations [34] and therefore reappraise whether the problem is indeed ‘trivial’ in the mind of the patient.

In addition to being contextual, empathy is also relational, requiring both an empathiser and an empathisee [33]. There is a strong sense within this data of students finding it easier to empathise with patients who are like them and who have problems like theirs’. This may reflect human nature, but it also presents a challenge to educators promoting the merits of viewing all patients with unconditional positive regard [35]. Shapiro has highlighted the limitations of reserving clinical empathy for selected in-groups and abandoning it for out-groups, including the stigmatised in society [27]. It is striking how many negative role model narratives in this study centred on patients with smoking, alcohol and other addictions. There is a hidden curriculum [3] for educators to uncover here. There may also be a role for education using the arts and humanities, which arguably open our horizons to a greater variety of social and cultural groups [36].

## Conclusion

In conclusion, our data lend support to the notion that clinical empathy is practiced both in cognitive and affective ways, with affective empathy being the deeper, more embracing form (Fig. 1). We have identified modifying factors, including barriers which either militate against empathy or promote cognitive over affective empathy, and chief amongst these is the pressure of time, which is currently under researched. We have also identified that medical communication teaching and OSCE assessment place an inherent emphasis on the empathic statement as the means to deliver empathy, despite students themselves preferring non-verbal approaches. Furthermore, this drive towards the empathic statement leaves students uncomfortable about feeling the need to force empathic statements at times when they are not feeling them—essentially practising empathy is a less than sincere way. We have termed this discomfort empathic dissonance, a novel concept in medical educational, and one which warrants further study. For those looking for practical implications, based on our findings, we propose five key practice points for educators (Table 3).

**Table 3:** Recommendations for educators

Recommendations for educators	
• When teaching empathy, include its affective as well as its cognitive components—for example encourage students to discuss their emotional reactions to patients with potentially upsetting presentations.	
• Encourage students to follow positive empathy role modelling and, of equal importance, to reflect on ‘how not to do it’ when they see negative role modelling.	
• Teach the limitations as well as the merits of the empathic statement, remind students of the importance of non-verbal communication.	
• Counter the problem of empathic dissonance—don’t press students into making empathic statements that are insincere and don’t assess empathy in every OSCE station.	
• Encourage students to explore the patient’s perspective before deciding if a complaint is ‘trivial’.	
